# Structural and pharmacological basis for the induction of mitochondrial biogenesis by formoterol but not clenbuterol

**DOI:** 10.1038/s41598-017-11030-5

**Published:** 2017-09-05

**Authors:** Robert B. Cameron, Yuri K. Peterson, Craig C. Beeson, Rick G. Schnellmann

**Affiliations:** 1 Department of Pharmacology and Toxicology, College of Pharmacy, University of Arizona, Tucson, AZ 85721 USA; 2 Department of Drug Discovery and Biomedical Sciences, College of Graduate Studies, Medical University of South Carolina, Charleston, SC 29425 USA; 3 Department of Drug Discovery and Biomedical Sciences, College of Pharmacy, Medical University of South Carolina, MSC139, 70 President St., Charleston, SC 29425-8906 USA

**Keywords:** Cell signalling, Receptor pharmacology

## Abstract

Mitochondrial dysfunction is associated with numerous acute and chronic degenerative diseases. The beta-2 adrenergic receptor (β_2_AR) agonist formoterol induces mitochondrial biogenesis (MB), but other β_2_AR agonists, such as clenbuterol, do not. We sought to identify the MB signaling pathway of formoterol and the differences in signaling between these two ligands that result in the differential induction of MB. While formoterol and clenbuterol increased cAMP, only formoterol increased the phosphorylation of Akt and its downstream target eNOS. The increase in Akt phosphorylation was Gβγ- and PI3K-dependent, and the increase in eNOS phosphorylation was Gβγ- and Akt-dependent. Only formoterol increased cGMP. Formoterol induced MB as measured by increases in uncoupled cellular respiration and PGC-1α and NDUFS1 mRNA expression and was blocked by inhibitors of Gβγ, Akt, NOS, and soluble guanylate cyclase. To identify distinct receptor-ligand interactions leading to these differences in signaling, we docked formoterol and clenbuterol to six structures of the β_2_AR. Compared to clenbuterol, the methoxyphenyl group of formoterol interacted more frequently with V114 and F193, while its formamide group interacted more frequently with C191. These data indicate that the unique structural features of formoterol allow it to interact with the β_2_AR to activate the Gβγ-Akt-eNOS-sGC pathway to induce MB.

## Introduction

Mitochondria play numerous roles in cellular homeostasis, including energy metabolism, synthesis of key biomolecules, regulation of reactive oxygen species, and apoptosis^1^. However, in disease states, dysfunctional mitochondria lead to metabolic defects and subsequent derangements in survival^2^, proliferation^3^, and differentiation^4–7^. One therapeutic strategy to treat mitochondrial dysfunction is the induction of mitochondrial biogenesis (MB)^8, 9^. By generating new mitochondria, MB increases cellular respiration and ATP, reduces pathologic oxidative stress, and promotes cell repair and regeneration^10, 11^.

A number of signaling molecules have been shown to induce MB, including transcription factors, kinases, cyclic nucleotides, and G protein-coupled receptors (GPCRs)^9^. In particular, GPCRs are attractive targets for the identification of therapeutics that induce MB because GPCR ligands represent numerous clinically approved receptor agonists^12^.

Previous work in our laboratory identified formoterol, a beta-2 adrenergic receptor (β_2_AR) agonist, as a potent and efficacious inducer of MB *in vitro* and *in vivo*^13^. Furthermore, in a mouse model of bilateral ischemic reperfusion-induced acute kidney injury formoterol stimulated MB with increased mitochondrial proteins and accelerated recovery of renal function^14^. Based on the success of formoterol, other β_2_AR agonists were screened for induction of MB. Although several agonists were able to induce MB similar to formoterol, several other agonists, including clenbuterol, were unable to induce MB at any concentration^15^. These data suggest that a subset of biogenic β_2_AR agonists modulates distinct signaling pathways from non-biogenic β_2_AR agonists to induce MB.

Because both formoterol and clenbuterol, a non MB inducer, are selective β_2_AR agonists^16^, we sought to identify the differences in signaling between the two agonists in primary cultures of renal proximal tubule cells (RPTCs) and the signaling pathway responsible for formoterol-induced MB. Furthermore, we explored their chemical differences to identify key functional groups and structural differences that result in their differing abilities to induce MB. We found that formoterol, but not clenbuterol, activates the Gβγ-Akt-eNOS-sGC signaling pathway and that this pathway is necessary for the transcriptional and functional changes associated with formoterol-induced MB. Molecular modeling showed that formoterol stretches further across the binding pocket than clenbuterol, allowing for more frequent interactions with V114, F193, and C191 at specific chemical features. Additionally, the methoxyphenyl and formamide groups displayed distinct interaction fingerprints with the β_2_AR that may lead to the activation of Gβγ-dependent signaling.

## Results

### Both formoterol and clenbuterol increase cAMP accumulation

**Figure 1:**
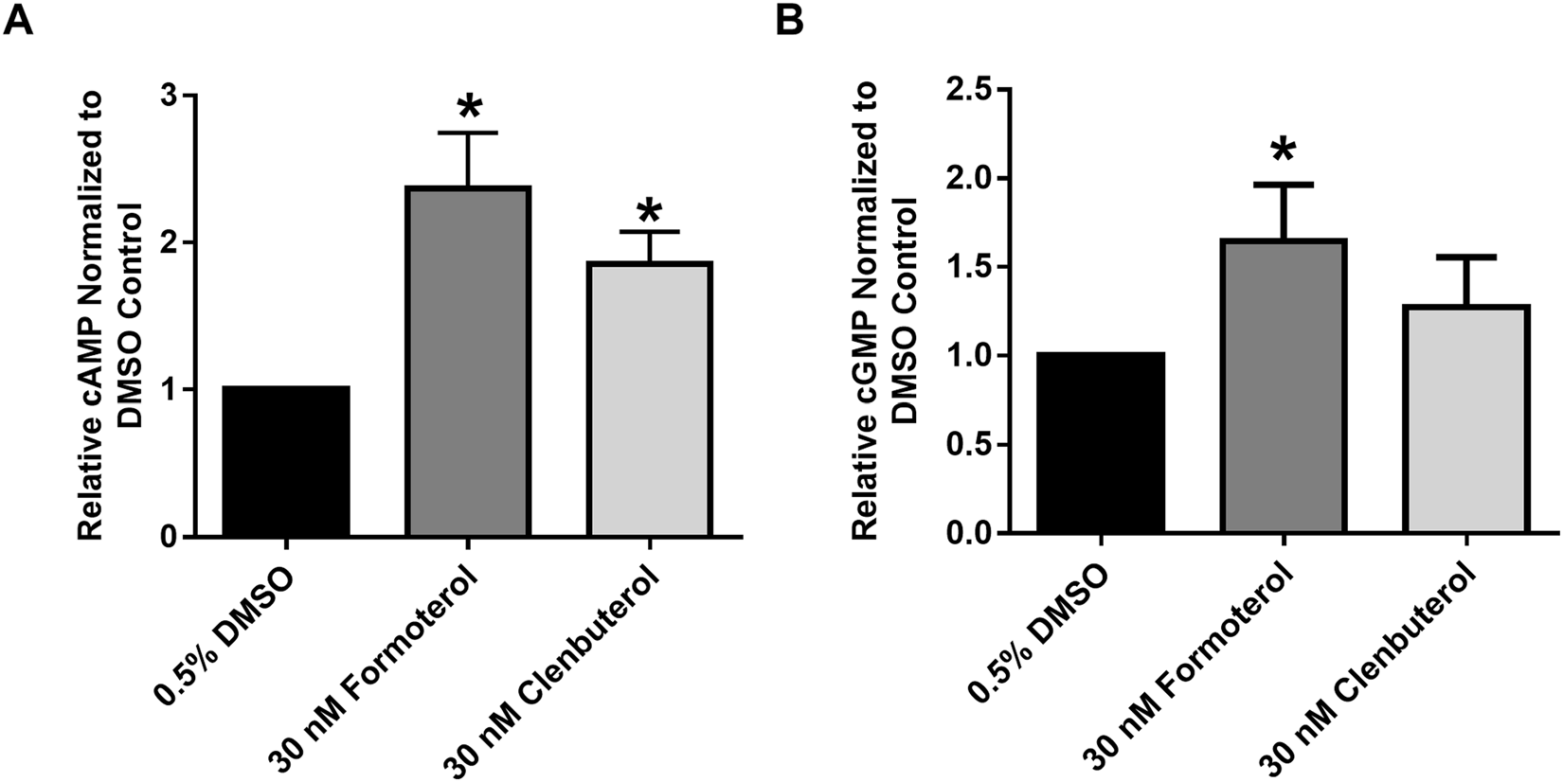
Formoterol and clenbuterol increase cAMP (**A**, N = 3) but only formoterol increases cGMP (**B**, N = 6) in RPTC. Levels of cAMP/cGMP were measured 1 h following treatment with formoterol or clenbuterol. Mean + SEM. ^*^p < 0.05, Wilcoxon signed rank test.

The β_2_AR couples to the stimulatory G protein Gα_s_ and the inhibitory G-protein Gα_i_, both of which affect the activity of adenylate cyclase and therefore the accumulation of cAMP. To assess the effects of formoterol and clenbuterol on cAMP accumulation, RPTC were co-treated for 1 h with 30 nM formoterol or 30 nM clenbuterol in the presence of 100 μM IBMX, a phosphodiesterase inhibitor to prevent cyclic nucleotide degradation. These concentrations ensure selective activation of the β_2_AR while also exerting the previously observed effects on MB. Maximal cAMP accumulation occurs at 1 h (data not shown)^15^. Both formoterol and clenbuterol increased cAMP relative to vehicle controls (Fig. 1A). Because there was no difference in cAMP accumulation between the two compounds, and previous work showed that cAMP does not produce MB in RPTC, we concluded that β_2_AR is functioning normally in RPTC with respect to cAMP production but that cAMP is not necessary for β_2_AR-mediated MB in RPTC.

### Formoterol, but not clenbuterol, increases Akt phosphorylation in a Gβγ-PI3K-dependent manner

**Figure 2:**
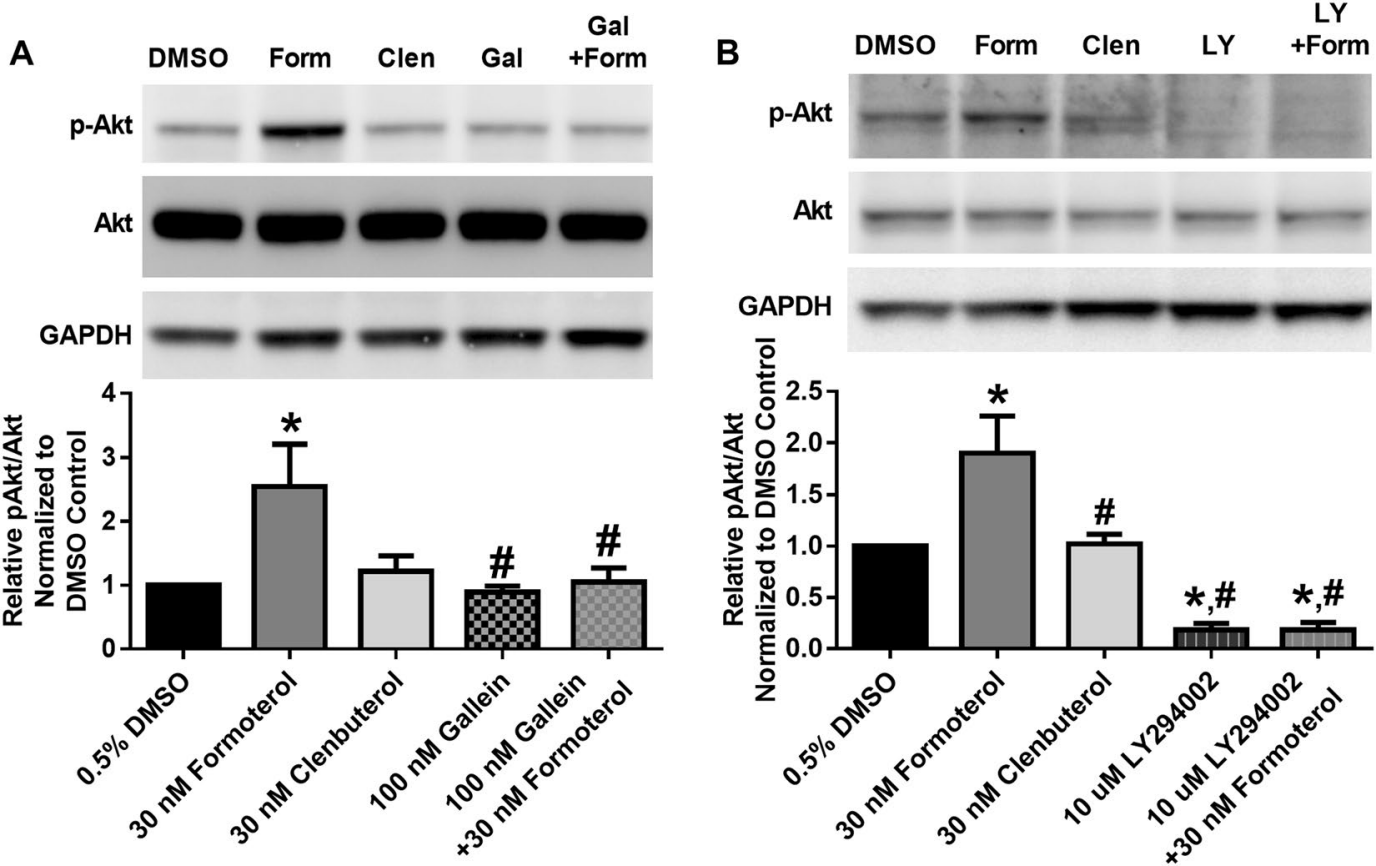
Formoterol, but not clenbuterol, activates Akt in a Gβγ-PI3K-dependent manner in RPTC. pAkt was measured following 30 min of formoterol (Form) or clenbuterol (Clen) in the presence and absence of the Gβγ inhibitor gallein (Gal)(A, N = 4) or the PI3K inhibitor LY294002 (LY)(B, N = 4–5). Images are cropped to show only relevant bands. Full sized images are available in Supplemental Figure 3. Mean + SEM. ^*^p < 0.05 vs. DMSO, #p < 0.05 vs. formoterol, one-way ANOVA with Sidak’s multiple comparison test.

In addition to their roles in the modulation of cAMP, both Gα_s_ and Gα_i_ release the Gβγ heterodimer. To assess the role of Gβγ, we measured Akt phosphorylation 30 min following treatment with formoterol or clenbuterol. This time point represents the earliest time point at which elevated Akt phosphorylation could be detected (data not shown). Formoterol increased Akt phosphorylation while clenbuterol did not (Fig. 2). Pretreatment with the Gβγ inhibitor gallein^17^ attenuated formoterol-induced Akt phosphorylation (Fig. 2A), as did pretreatment with the phosphatidylinositol-4,5-biphosphate 3-kinase (PI3K) inhibitor LY294002 (Fig. 2B)^18^. These data indicate that formoterol, but not clenbuterol, increases Akt phosphorylation in a Gβγ-PI3K-dependent manner.

### Formoterol, but not clenbuterol, increases eNOS phosphorylation in a Gβγ-Akt-dependent manner

**Figure 3:**
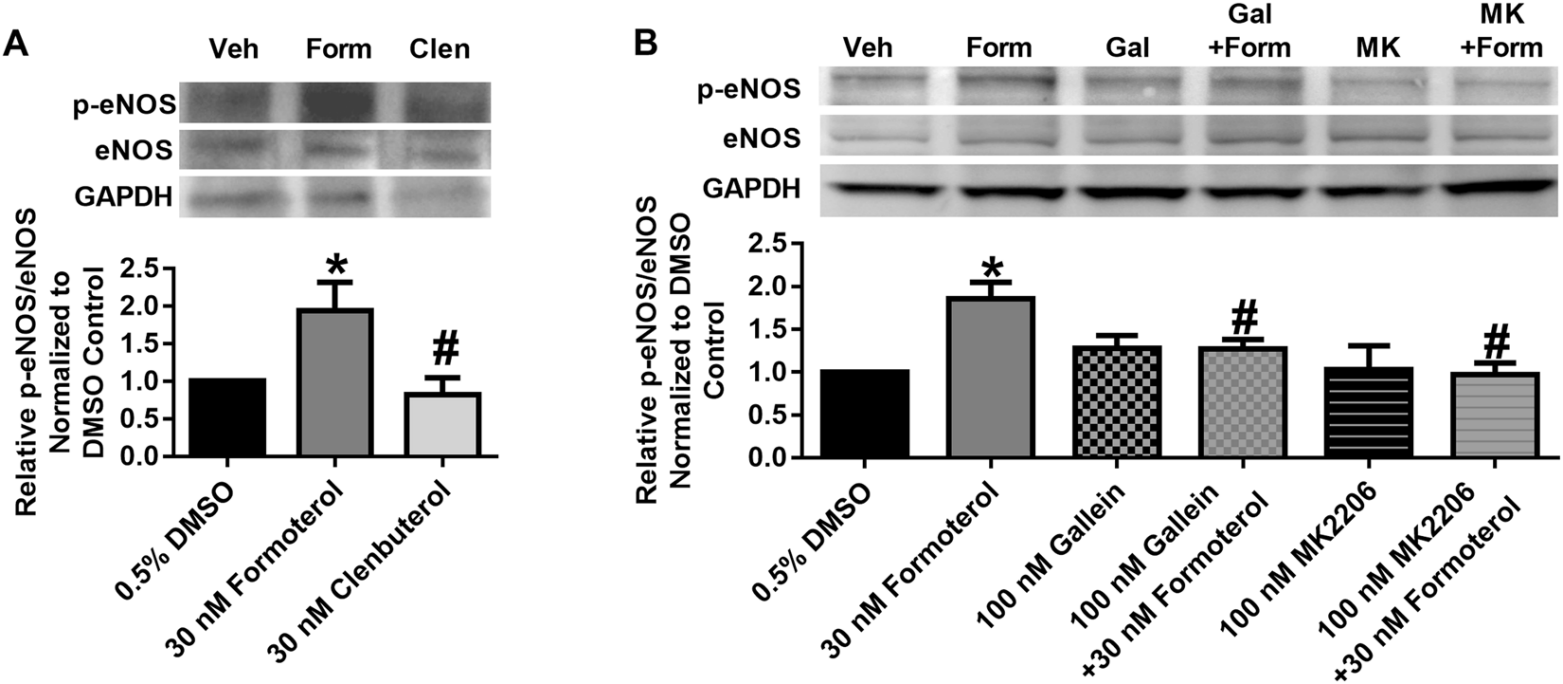
Formoterol (Form), but not clenbuterol (Clen), activates eNOS in a Gβγ- and Akt-dependent manner in RPTC. (**A**) p-eNOS was measured following 1 h of formoterol or clenbuterol. N = 5–6. (**B**) Phosphorylation of eNOS was measured following 30 min of treatment with formoterol in the presence or absence of the Gβγ inhibitor gallein (Gal) or the Akt inhibitor MK2206 (MK). N = 7–10. Images are cropped to show only relevant bands. Full sized images are available in Supplemental Figure 3. Mean + SEM. ^*^p < 0.05 vs. DMSO, ^#^p < 0.05 vs formoterol, one-way ANOVA with Sidak’s multiple comparison test.

Among the downstream targets of Akt is endothelial nitric oxide synthase (eNOS)^19^. Upon phosphorylation at S1177, eNOS is activated and increases NO generation. Because NO and NO-dependent signaling have been implicated in MB, we treated RPTC with formoterol and clenbuterol for 1 h to determine differences in eNOS phosphorylation. Formoterol increased eNOS phosphorylation relative to vehicle control, while clenbuterol did not affect eNOS phosphorylation (Fig. 3A).

To determine the role of the Gβγ-Akt pathway in formoterol-induced eNOS phosphorylation, RPTC were pretreated with the Gβγ inhibitor gallein or the Akt inhibitor MK2206 followed by treatment with formoterol for 1 h. The allosteric Akt inhibitor MK2206 was used because it decreases Akt phosphorylation, thereby confirming that sufficient Akt inhibition had occurred^20^. Both gallein and MK2206 attenuated formoterol-induced eNOS phosphorylation (Fig. 3B), indicating that formoterol, but not clenbuterol, activates the Gβγ-Akt-eNOS signaling pathway.

### Formoterol, but not clenbuterol, increases cGMP accumulation

One of the major targets of NO is soluble guanylate cyclase (sGC). To examine any differences in cGMP accumulation, RPTC were treated with formoterol or clenbuterol in the presence of 100 μM IBMX for 1 h, and cGMP was determined by ELISA. Formoterol, but not clenbuterol, increased levels of cGMP relative to vehicle control (Fig. 1B).

### Formoterol increases maximal respiration in a Gβγ-Akt-NOS-sGC-dependent manner

**Figure 4:**
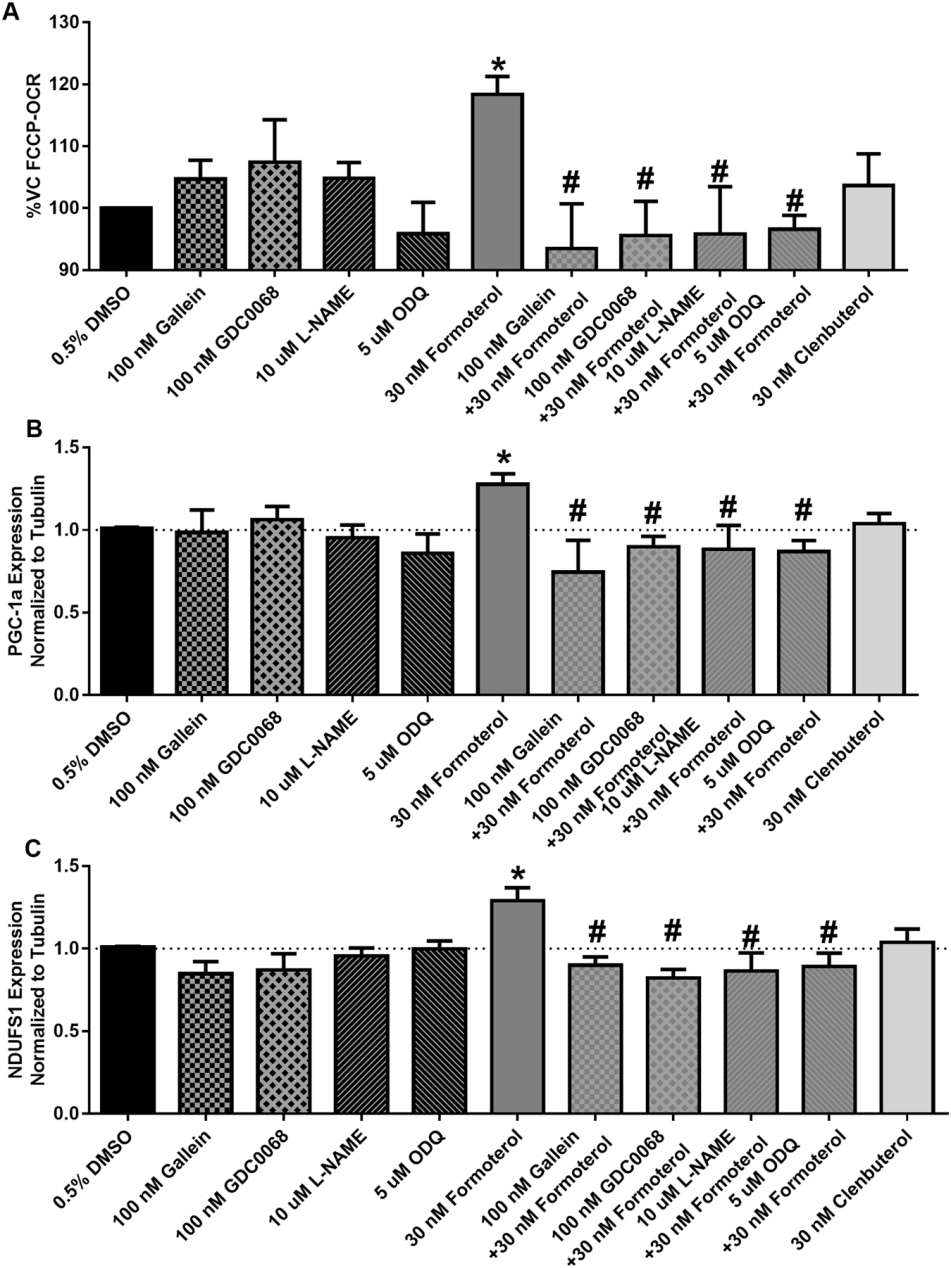
Formoterol, but not clenbuterol, induces MB in a Gβγ-Akt-NOS-sGC-dependent manner. (**A**) FCCP-OCR (N = 3–9), (**B**) PGC1α mRNA (N = 3–10), and (**C**) NDUFS1 mRNA (N = 4–10) expression was measured 24 h following treatment with formoterol in the presence or absence of the Gβγ inhibitor gallein, the Akt inhibitor GDC0068, the NOS inhibitor L-NAME, and the sGC inhibitor ODQ. Mean + SEM. ^*^p < 0.05 vs DMSO, ^#^p < 0.05 vs. Formoterol, one-way ANOVA with Sidak’s multiple comparison test.

Having shown that formoterol but not clenbuterol activates the Gβγ-Akt-eNOS-sGC pathway, we assessed the role of this pathway in formoterol-induced increases in FCCP-OCR, a measure of MB. RPTC were pretreated with the Gβγ-inhibitor gallein, the Akt inhibitor GDC0068, the NOS inhibitor L-NAME, and the sGC inhibitor ODQ. The orthosteric Akt inhibitor GDC0068 was used due to its greater potency and lack of isoform selectivity^21^. RPTC were then treated with formoterol or clenbuterol for 24 h, and FCCP-OCR consumption was measured. This time point was chosen based on previous studies identifying that formoterol induces MB at 24 h^13, 15^. Formoterol alone increased FCCP-OCR, in agreement with previous studies (Fig. 4A)^13, 15^. Pretreatment with gallein, GDC0068, L-NAME, and ODQ attenuated formoterol-induced increases in FCCP-OCR, indicating that formoterol-induced MB occurs in a Gβγ-Akt-NOS-sGC-dependent manner. Clenbuterol had no effect on FCCP-OCR.

### Formoterol, but not clenbuterol, increases mRNA expression of PGC-1α and NDUFS1 in a Gβγ-Akt-NOS-sGC-dependent manner

MB requires the integrated transcription of multiple genes. To assess the effects of the Gβγ-Akt-eNOS-sGC pathway on the expression of genes associated with MB, RPTC were pretreated with gallein, GDC0068, L-NAME, and ODQ, followed by treatment with formoterol or clenbuterol for 24 h. RNA expression of peroxisome proliferator activated receptor gamma coactivator-1α (PGC-1α) and NADH-ubiquinone oxidoreductase core subunit S1 (NDUFS1) was assessed using RT-qPCR. Formoterol alone caused a small but significant increase in PGC-1α and NDUFS1 (Fig. 4B,C), which was attenuated by pretreatment with gallein, GDC0068, L-NAME, and ODQ. These data indicate that formoterol also increases transcriptional markers of MB in a Gβγ-Akt-NOS-sGC-dependent manner. Importantly, clenbuterol did not increase the mRNA expression of PGC-1α or NDUFS1. These data were further confirmed by measuring mtDNA copy number, where formoterol, but not clenbuterol, increased mtDNA copy number at 24 h (Figure S1).

### Formoterol and clenbuterol have distinct interaction fingerprints with the β_2_AR

**Figure 5:**
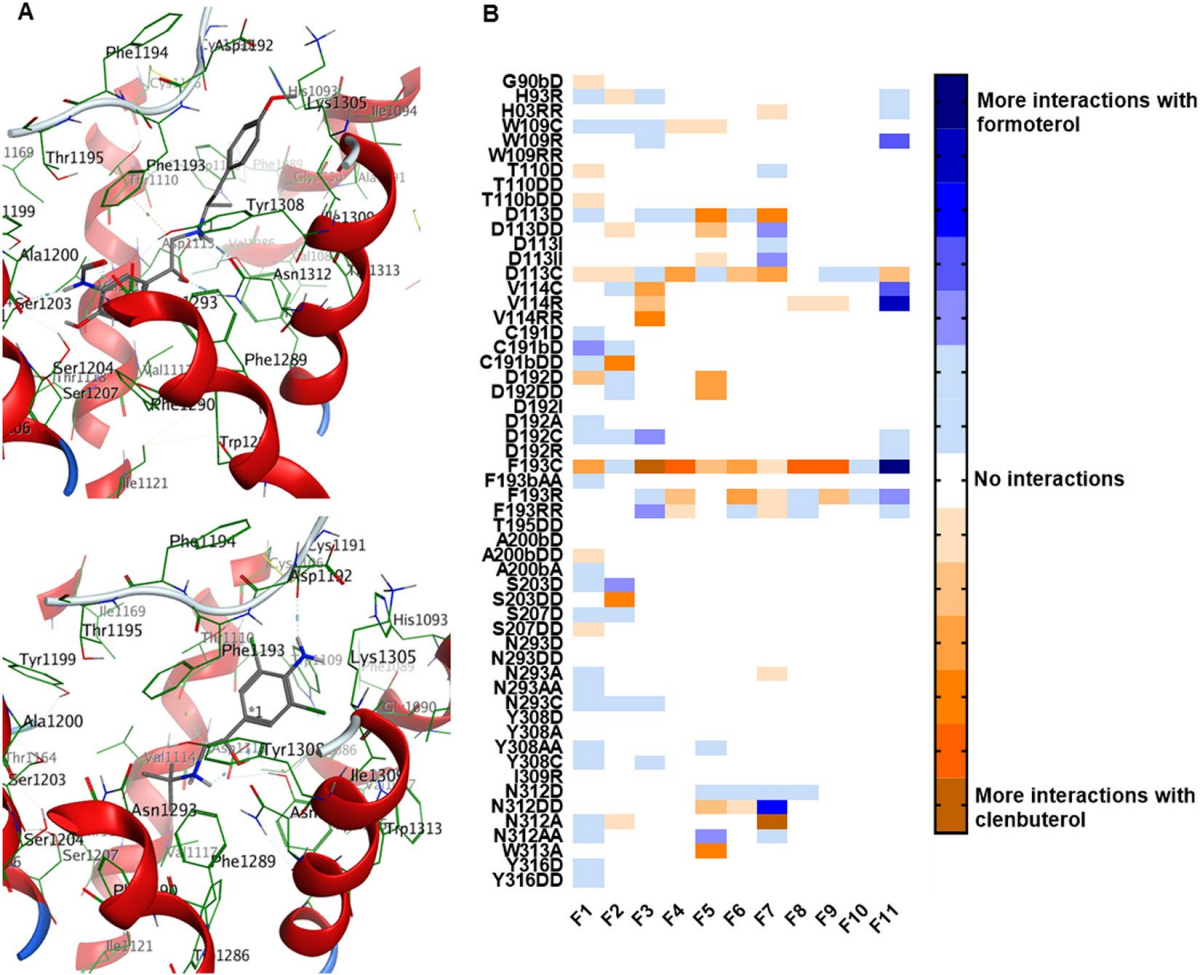
Formoterol and clenbuterol have distinct interaction fingerprints with the β_2_AR. (**A**) Representative poses of formoterol and clenbuterol in the 4LDO crystal structure. Formoterol’s methoxyphenyl group (top) allows it to extend further across the binding pocket than clenbuterol (bottom), allowing formoterol to simultaneously interact with TM3, TM5, and ECL2 and be in closer proximity to TM2. (**B**) Formoterol and clenbuterol were docked to inactive structures (3NYA, 3NY8, 5D5B) and active structures (4LDE, 4LDL, 4LDO) of the β_2_AR. From the top 30 poses, ligand interactions were matched with structural features F1-F11 and added for each crystal structure. More negative (orange) values correspond to a greater number of interactions with clenbuterol, while more positive (blue) values correspond to a greater number of interactions with formoterol. Interactions are displayed as Residue-interaction type (D- hydrogen bond donor, DD- strong hydrogen bond donor, I- ionic, II- strong ionic, A- hydrogen bond acceptor, AA- strong hydrogen bond acceptor, C- contact, R- arene, RR- strong arene). Residues followed by a b (e.g., G90b) indicate an interaction with the peptide backbone of the corresponding residue.

Due to the differences in signaling described above, we investigated differences in interactions of β_2_AR agonists with the receptor. In addition to formoterol and clenbuterol, other ligands with known efficacy for MB were considered, including fenoterol, ritodrine, and terbutaline^15^. Ligands were docked to three inactive structures of the β_2_AR (3NYA, 3NY8, 5D5B) and three active structures of the β_2_AR (4LDE, 4LDL, 4LDO). Due to its longer methoxyphenyl group, formoterol was able to extend across the binding pocket to be in proximity to TM2, TM3, ECL2, and TM5 compared to clenbuterol (Fig. 5A).

**Figure 6:**
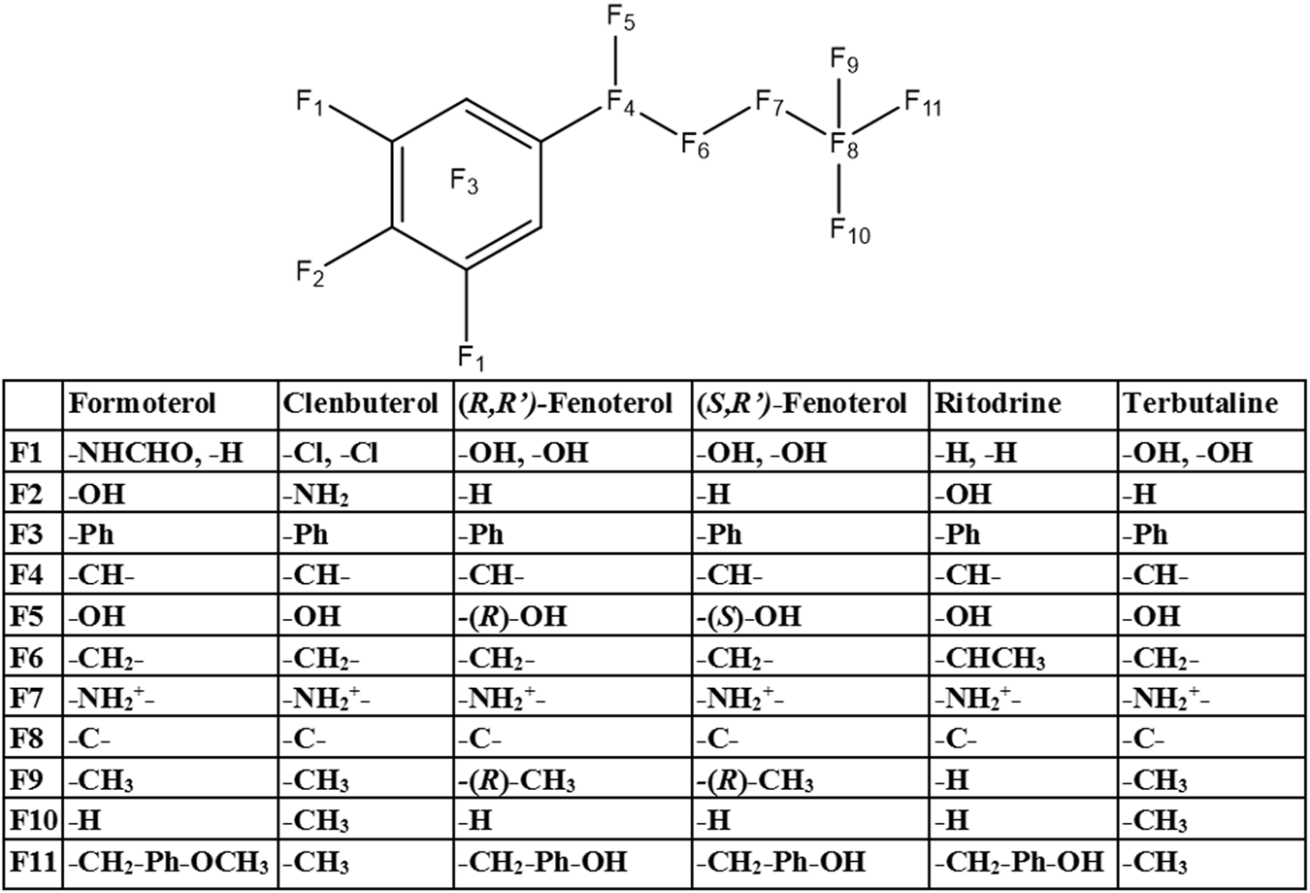
Dividing β_2_AR agonists into chemical groups reveals distinct structural features. Formoterol, clenbuterol, (R,R’)-fenoterol, (S,R’)-fenoterol, ritodrine, and terbutaline were divided into 11 chemical features (F1-F11). Ph- phenyl group.

To investigate the interactions of formoterol and clenbuterol with the β_2_AR with respect to distinct structural features, functional groups of the ligands were combined to form 11 structural features F1-F11 (Fig. 6), with the catecholamine pharmacophore represented by F1-F7 and the tail group represented by F8-F11. For each conformation generated by the docking simulations, all interactions between the receptor and the ligand were tabulated. Interactions between the ligand and each receptor amino acid were separated by residue, ligand feature, and type [i.e., contact (C), arene (R), hydrogen bond donor (D), hydrogen bond acceptor (A), and ionic (I)]. Interactions at each of the 11 structural features were added for all generated conformations. To identify interactions specific to biogenic β_2_AR agonists, the interactions of the compound with less efficacy for MB were subtracted from those of the compound with greater efficacy for MB (e.g., ΣInteractions_Formoterol_-ΣInteractions_Clenbuterol_). The resulting values were used to generate a heatmap for the interactions between the receptor and specific structural features of the ligand. Interaction pairs with more positive values (blue) indicated a greater importance for β_2_AR-mediated MB (Fig. 5B). Interaction pairs with more negative values (orange) indicate importance for stabilizing non-mitochondrially biogenic conformations of the β_2_AR. This analysis enables the identification of features of both the ligand and the receptor that distinguish biogenic and non-biogenic β_2_AR agonists.

As expected for two β_2_AR agonists with a shared pharmacophore, most of the interactions showed only minor preference for formoterol or clenbuterol, with several interactions occurring with the same frequency (Fig. 5B). Interactions between the backbone of C191 and F1 were more common for formoterol than clenbuterol, while the reverse was true for F2. Interestingly, interactions with both V114 and F193 were more common for clenbuterol over features F3-F10; however, at F11, interactions with V114 and F193 more commonly occurred with formoterol than clenbuterol. These data reveal a common pharmacophore and marked differences in the binding of formoterol and clenbuterol to the β_2_AR crystal structure and suggest that interactions between distinct β_2_AR residues and distinct ligand structural features must occur to activate the Gβγ-Akt-eNOS-sGC pathway and induce MB.

To confirm the importance of the interactions identified above, we repeated the ligand interaction analysis for other ligands previously tested for MB. Fenoterol is a β_2_AR agonist that induces MB in a manner similar to formoterol^15^. The (*R*,*R’*) enantiomer of fenoterol is a Gα_s_-biased ligand; however, the (*S,R’*) enantiomer activates both Gα_s_ and Gα_i_, potentially enabling it to activate Gβγ-dependent signaling pathways like formoterol^22^. Comparing (*S*,*R’*)-fenoterol to (*R,R’*)-fenoterol showed that the former was more likely to interact with V114 and F193 at F11 and engage in hydrogen bonding interactions with S203, S207, and the backbone of C191 (Figure S2A). Because these differences are similar to those between formoterol and clenbuterol, these data suggest that the (*S,R’*) enantiomer of fenoterol with a greater capacity to activate Gβγ-dependent signaling is more capable of inducing MB than the Gα_s_-biased (*R,R’*) enantiomer. Comparing (*S,R’*)-fenoterol to clenbuterol showed a similar interaction profile to formoterol vs. clenbuterol (Figure S2B). Interactions with F193 and V114 at F11 were again more common with (*S,R’*)-fenoterol, although interactions with V114 had a greater tendency to be aromatic than seen with formoterol. (*S,R’*)-fenoterol also had more hydrogen bonding interactions at F1, particularly with S207, S203, and the backbone of C191.

To confirm the importance of interactions at F11, we compared (*S,R’*)-fenoterol to ritodrine (Figure S2C). Ritodrine can induce MB at low but not high concentrations and is structurally similar to fenoterol with identical features F3, F4, F5, F7, F8, F10, and F11 (that is, differing only at F1, F2, F6, and F9)^15^. At F11, (*S,R’*)-fenoterol interacted more frequently with F193 than ritodrine. Because these ligands share a common feature F11 but differ in biogenic status, the interaction of F193 with F11 is important for β_2_AR-mediated MB.

To confirm the importance of interactions at F1, we compared terbutaline to clenbuterol (Figure S2D). Like ritodrine, terbutaline induces MB at low but not high concentrations^15^. Terbutaline is also structurally similar to clenbuterol, sharing identical features F3-F11 (that is, differing only at F1 and F2). At feature F1, terbutaline interacted more frequently with S203, S207, and N293. When considered with the interaction profiles of formoterol and (*S,R’*)-fenoterol, these data suggest that interactions with S203 and S207 at feature F1 are important for β_2_AR-mediated MB. Interestingly, terbutaline also interacted more frequently with F193 at F8, F19, F10, and F11 than clenbuterol, suggesting that the 2,5-hydroxyl substituted ring of terbutaline and fenoterol facilitates interactions with F193 at features F8-F11.

## Discussion

**Figure 7:**
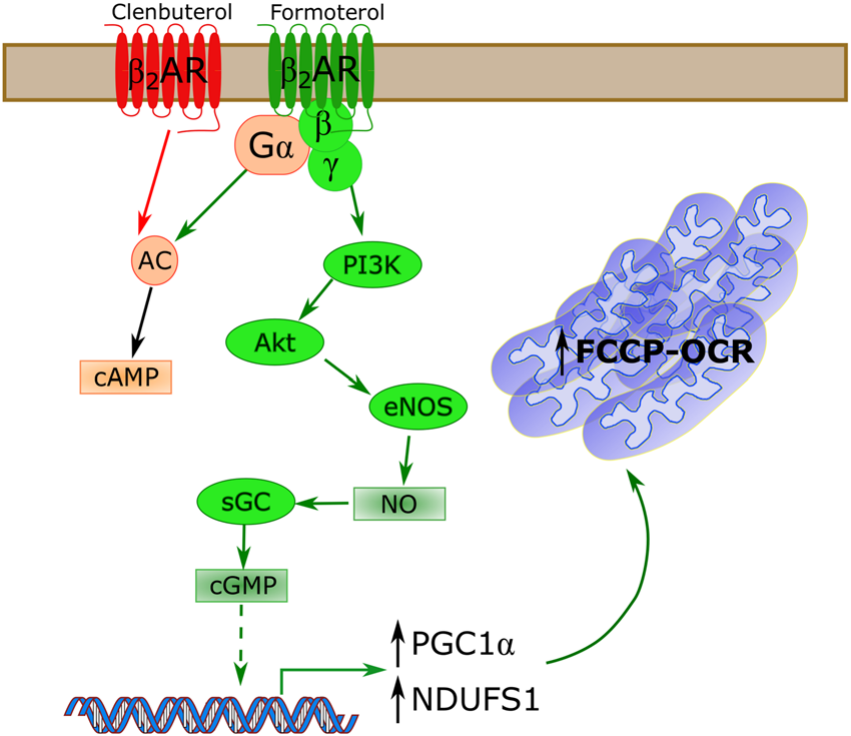
Formoterol, but not clenbuterol, induces MB in a Gβγ-Akt-NOS-sGC-dependent manner despite increased cAMP accumulation. Both formoterol and clenbuterol activate Gα_s_-dependent signaling to activate adenylate cyclase (AC) and promote cAMP accumulation. However, only formoterol activates the Gβγ-PI3K-Akt-eNOS-sGC pathway, and it is this pathway that is necessary for β_2_AR-induced MB in RPTC.

MB plays a vital role in regulating cellular metabolism, differentiation, and repair, and its pharmacologic induction has great therapeutic potential in a variety of disease states^8, 9^. Here, we show that in RPTC, formoterol, but not clenbuterol, activates the Gβγ-Akt-eNOS-sGC signaling pathway and that this pathway is necessary for formoterol-induced MB (Fig. 7). Importantly, these experiments were performed in metabolically competent primary cells that can better mirror *in vivo* signaling and metabolism compared to immortalized cell lines. Therefore, these data suggest a novel role of Gβγ-dependent signaling for GPCR-mediated MB in other non-renal tissues.

The Gβγ heterodimer is released from heterotrimeric G proteins following GPCR activation. Gβγ is primarily activated by the G_i/o_ family of G proteins; however, other G protein families, including G_s_, release Gβγ^23^. Gβγ heterodimers have varied effects on signal transduction, including PI3K activation, adenylate cyclase stimulation, adenylate cyclase inhibition, MAPK activation, and GRK activation, depending on their constituent Gβ and Gγ subunits^24^. GPR43 was shown to signal through Gβγ and its activation by acetate induced MB^25^, but the lack of inhibitor studies meant that a causal link between Gβγ and MB was not established. By pretreating cells with the Gβγ inhibitor gallein, we identified that Gβγ-dependent signaling is a key pathway for GPCR-mediated MB.

It is important to note that the present study does not determine whether or not Gβγ directly activates the PI3K-Akt pathway. In addition to the activation of the Ras-PI3K-Akt pathway, Gβγ can also facilitate GRK2 recruitment to the receptor and lead to arrestin-dependent signaling. Arrestins are also capable of activating Akt in a PI3K-Src-dependent mechanism^26^. However, given that the arrestin-biased agonist isoetharine is unable to induce MB^15^, this mechanism is unlikely.

Prolonged activation of Akt enhances cellular survival but can lead to a decrease in mitochondrial function^27–29^. In contrast, acute activation of Akt is responsible for the effects of multiple inducers of MB, including (-)-epicatechin and erythropoietin^30, 31^. For such compounds, the role of Akt seems to be limited to the phosphorylation of eNOS, leading to NO generation. Nitric oxide is a potent inducer of MB *in vitro* and *in vivo* through the activation of sGC and subsequent cGMP accumulation^32^. However, because NO is a free radical, sustained NOS activation can increase oxidative and proteotoxic stress and can inhibit complex I of the electron transport chain^33, 34^. Thus, both the signaling cascade activated by a compound and the duration of that signaling contribute to the therapeutic potential of inducers of MB.

Interestingly, there are conflicting data regarding the role of Gβγ in acute organ injury, which is frequently characterized by mitochondrial dysfunction. Inhibition of Gβγ by gallein inhibits RPTC proliferation and thereby exacerbates ischemic acute kidney injury^35^. In contrast, gallein also prevents inflammatory cell infiltration^17^, leading to enhanced recovery following ischemic acute kidney injury^36^. Although neither study assessed mitochondrial activity, both regeneration following injury and inflammatory cell chemotaxis are enhanced by increases in mitochondrial activity^37, 38^.

The β_2_AR is a prototypical class A GPCR and has been extensively studied for its role in cellular signaling and the structural features that enable such signaling. This study is the first to examine the receptor-ligand interactions that distinguish mitochondrial biogenic β_2_AR agonists from non-biogenic β_2_AR agonists. As expected for two agonists of the β_2_AR, formoterol and clenbuterol have a common pharmacophore, and many of the interactions showed little preference for formoterol or clenbuterol. Nonetheless, the structural dissimilarities of the two compounds led to several distinct receptor-ligand interactions. In particular, interactions with V114 and F193 tended to occur more frequently at the methoxyphenyl group on formoterol, while C191 and its peptide backbone interacted more frequently with the formamide group of formoterol. To enable these interactions to occur, formoterol binds to the β_2_AR in a conformation that places it near the deeper pocket residues of TM3 and TM5 as well as near the shallower residues of TM2, ECL2, and ECL4. Our observation of the proximity to ECL2 and ECL4 with formoterol is in agreement with NMR studies showing that formoterol weakens the ionic interaction between D192 and K305^39^. Furthermore, ECL2 flexibility is important to ligand activity at the β_2_AR and for other GPCRs, such as the D_2_ dopamine receptor^40^.

Modeling interactions of other β_2_AR agonists with the receptor further supported the role of a subset of receptor ligand interactions in stabilizing conformations of the β_2_AR that lead to MB. When compared to the non-biogenic agonist clenbuterol or the partial agonist of MB ritodrine, the hydroxyphenyl group (F11) of (*S,R’*)-fenoterol was more likely to engage in contact interactions with F193 and hydrogen bond donor interactions with C191 and its backbone. Additionally, its 3,5-hydroxyl groups (F1) were more likely to act as hydrogen bond donors for S203 and S207. These results are in agreement with previous docking studies of fenoterol with the β_2_AR^41^. Similarly, when compared to clenbuterol, the partial agonist of MB terbutaline had more contact interactions between its *tert*-butyl group (F8-F11) and F193 and hydrogen bond donor interactions between its 3,5-hydroxyl groups (F1) and S203, S207, and N293. Previous docking studies with terbutaline identified interactions between terbutaline and F193 but not between S203, S207, or N293^42^. However, those studies generated homology models of the rat β_2_AR from 2RH1, which represents an inactive conformation of the receptor^43^, while our study employed both active and inactive structures of the β_2_AR.

Previous NMR studies have shown that formoterol and clenbuterol engage different conformations of the β_2_AR^44^, particularly regarding the conformational shift of TM6. Indeed, numerous studies using NMR and mass spectrometry have shown that functionally similar agonists can effect distinct active conformations of the receptor^45–47^. Additionally, the conformational flexibility of the β_2_AR allows for multiple “active” conformations that may lead to differences in effector coupling. Among its active conformations, formoterol may stabilize a set of biogenic β_2_AR conformations that are thermodynamically unfavorable for clenbuterol.

In conclusion, this study identified a distinct signaling pathway activated by the mitochondrial biogenic β_2_AR agonist formoterol but not by the non-biogenic β_2_AR agonist clenbuterol in metabolically competent primary cells. This Gβγ-Akt-eNOS-sGC pathway is necessary for the transcriptional and functional changes associated with MB. We also identified distinct structural features and ligand interactions that may allow formoterol to activate this pathway. Together, these data can facilitate the development of novel β_2_AR agonists that selectively stimulate the Gβγ-Akt-eNOS-sGC pathway to induce MB and recovery from acute and chronic degenerative diseases.

## Methods

### Reagents

Anti-GAPDH antibody was purchased from Fitzgerald Antibodies (Acton, MA) and was used at a dilution of 1:1,000. Anti-phospho-Akt (Ser473), anti-Akt, and anti-phospho-eNOS (Ser1177) antibodies were purchased from Cell Signaling (Danvers, MA) and were all used at a dilution of 1:1,000. Anti-eNOS antibody (1:500 dilution), anti-mouse IgG (1:10,000 dilution), and anti-rabbit IgG (1:2,000 dilution) antibodies were purchased from Abcam (Cambridge, MA). MK2206 and GDC0068 were purchased from SelleckChem (Houston, TX). Gallein, LY294002, L-NAME, and ODQ were purchased from Tocris (Ellisville, MO). All other chemicals were purchased from Sigma (St. Louis, MO).

### Isolation and culture of proximal tubule cells

Female New Zealand white rabbits (1.5–2.0 kg) were purchased from Charles River Laboratories (Wilmington, MA). RPTCs were isolated via the iron oxide perfusion method, and RPTCs were cultured under improved conditions as described previously^48, 49^. Three days after initial playing, dedifferentiated RPTCs were trypsinized and replated on XF-96 polystyrene culture microplates (Seahorse Bioscience, North Billerica, MA) at a density of 18,000 cells/well and were maintained at 37 °C for 3 days before pharmacological manipulation. For other RPTC experiments, isolated renal proximal tubules were plated in 35 mm dishes and used at confluence 6 days after initial plating. All experiments were carried out in accordance with the recommendations in the Guide for the Care and Use of Laboratory Animals of the National Institutes of Health. All procedures were approved by the Institutional Animal Care and Use Committees of the Medical University of South Carolina and the University of Arizona, and appropriate efforts were made to reduce animal suffering.

### Measurement of oxygen consumption

The oxygen consumption rate (OCR) of RPTCs was measured using the Seahorse Bioscience XF-96 Extracellular Flux Analyzer as previously described^50^. RPTCs in 96-well assay plates were treated with vehicle control (dimethylsulfoxide (DMSO), <0.5%) or with experimental compounds. Basal OCR was measured, followed by injection of 10 μM carbonyl cyanide 4-(trifluoromethoxy)phenylhydrazone (FCCP) to allow for the measurement of uncoupled OCR (FCCP-OCR), a marker of MB.

### Protein isolation and immunoblotting

Freshly isolated RPTCs were suspended in protein lysis buffer (1% Triton X-100, 150 mM NaCl, and 10 mM Tris-HCl, pH 7.4; 1 mM EDTA; 1 mM EGTA; 2 mM sodium orthovanadate; 0.2 mM phenylmethylsulfonyl fluoride; 1 mM HEPES, pH 7.6) containing protease inhibitors and phosphatase inhibitors (Sigma-Aldrich, St. Louis, MO). Following sonication, protein was quantified using a bicinchoninic acid assay, subjected to SDS-PAGE, transferred onto nitrocellulose membranes, and incubated with primary and secondary antibodies. Membranes were detected using chemiluminescence and processed using ImageJ (NIH, Bethesda, MD) software.

### Nucleic acid isolation and quantitative polymerase chain reaction

To measure RNA expression, RPTC were scraped in TRIzol (Life Technologies, Grand Island, NY), and RNA was isolated using a phenol-based centrifugation method. cDNA was reversed transcribed from 5 μg RNA using the iScript Advanced cDNA Synthesis Kit (BioRad, Hercules, CA), diluted 1:10, and 5 μL added to a real-time SYBR green quantitative polymerase chain reaction master mix (BioRad). Changes in gene expression were calculated based on the Δ-Δ threshold cycle method. The following primers were used: PGC1α forward (AGGAAATCCGAGCCGAGCTGA), PGC1α reverse (GCAAGACGGAGACACATCAAA), NDUFS1 forward (AGATGATTTGGGAACAACAG), NDUFS1 reverse (TAGGGCTTAGAGGTTAGAGC), tubulin forward (CTCTCTGTCGATTACGGCAAG), and tubulin reverse (TGGTGAGGATGGAGTTGTAGG).

To measure mtDNA copy number, RPTC were scraped in phosphate buffered saline, and DNA was extracted using the DNeasy Blood and Tissue kit (QIAGEN, Valencia, CA). PCR products were amplified from 50 ng of cellular DNA using a real-time SYBR green quantitative polymerase chain reaction master mix (BioRad). For estimation of mtDNA, the NADH dehydrogenase subunit 6 (ND6) gene was used and normalized to tubulin. The following primers were used: ND6 forward (ACTGCGATGGCAACTGAGGAGTAT), ND6 reverse (ACCATAACTATACAACGCCGCCAC), tubulin forward (CTCTCTGTCGATTACGGCAAG), and tubulin reverse (TGGTGAGGATGGAGTTGTAGG).

### Measurement of cyclic nucleotides

RPTCs in 35-mm dishes were treated with vehicle control or the compound of interest for 1 h. RPTCs were then harvested according to the manufacturer’s protocol (Cayman Chemical, Ann Arbor, MI). Levels of cAMP and cGMP were measured using a commercially available enzyme-linked immunosorbent assay kit. Values were normalized to protein as quantified by a bicinchoninic acid assay followed by normalization to vehicle control for each biological replicate.

### Molecular modeling

Modeling, simulations, and visualizations were performed using MOE (Molecular Operating Environment) version 2015.1001 (Chemical Computing Group). The structural files used as input for analysis and docking simulations were PDB codes 3NYA, 3NY8, 4LDE, 4LDL, 4LDO, and 5D5B. Before analysis and simulations, all atoms and molecules other than the receptor and the ligand were removed. The receptor and all ligands were protonated at pH 7.4 in MOE. Initial placement calculated 30 poses per molecule using triangle matching placement with London dG scoring. All 30 poses were then refined using induced fit with London dG scoring. Ligand interactions were assessed using the protein-ligand interaction fingerprint (PLIF) function in MOE. To score interaction frequencies, interactions with individual atoms were tabulated. Interactions with the active ligand formoterol were assigned a value of 1, while interactions with the inactive ligand clenbuterol were assigned a value of -1, and interactions for all poses across all crystal structures were added to generate overall ligand interaction frequencies at structural features.

### Statistical analysis

Data are expressed as means ± S.E.M. (N ≥ 3) for all experiments. Each N represents a biological replicate. Multiple comparisons of normally distributed data were analyzed by one-way analysis of variance, as appropriate. Single comparisons were analyzed with the Wilcoxon signed rank test where appropriate. The criterion for statistical differences was p < 0.05 for all comparisons.

## Electronic supplementary material

Supplementary Information

## Data Availability

The data sets generated and/or analyzed from the present study are available from the corresponding author at reasonable request.
