# Author Correction: Structural and pharmacological basis for the induction of mitochondrial biogenesis by formoterol but not clenbuterol

**DOI:** 10.1038/s41598-019-42463-9

**Published:** 2019-05-01

**Authors:** Robert B. Cameron, Yuri K. Peterson, Craig C. Beeson, Rick G. Schnellmann

**Affiliations:** 1 Department of Pharmacology and Toxicology, College of Pharmacy, University of Arizona, Tucson, AZ 85721 USA; 2 Department of Drug Discovery and Biomedical Sciences, College of Graduate Studies, Medical University of South Carolina, Charleston, SC 29425 USA; 3 Department of Drug Discovery and Biomedical Sciences, College of Pharmacy, Medical University of South Carolina, MSC139, 70 President St., Charleston, SC 29425-8906 USA

Correction to: *Scientific Reports* 10.1038/s41598-017-11030-5, published online 05 September 2017

In the original version of this Article, Yuri K. Peterson was inadvertently omitted from the author list. This has been corrected in the PDF and HTML versions of the Article, and in the accompanying Supplementary Information file.

