# Supplementary material for: Structural and pharmacological basis for the induction of mitochondrial biogenesis by formoterol but not clenbuterol
Source: Sci Rep. 2017 Sep 5;7:10578. doi: 10.1038/s41598-017-11030-5 (PMC5585315; doi:10.1038/s41598-017-11030-5)
Supplement: Supplementary file 1 — Supplementary Information [file 41598_2017_11030_MOESM1_ESM.docx]

**Supplemental Information**

**Structural and pharmacological basis for the induction of mitochondrial biogenesis by formoterol and not clenbuterol**

Robert B. Cameron, Yuri K. Peterson, Craig C. Beeson, Rick G. Schnellmann

**Supplementary Figure S1.** Formoterol, but not clenbuterol, increases mtDNA copy number. RPTC were treated with 30 nM formoterol and 30 nM clenbuterol for 24 h. mtDNA copy number was estimated using qPCR for the mitochondrial gene ND6 relative to the nuclear gene tubulin. Data are presented as mean + SEM. N=4, *-p<0.05, Mann-Whitney test.


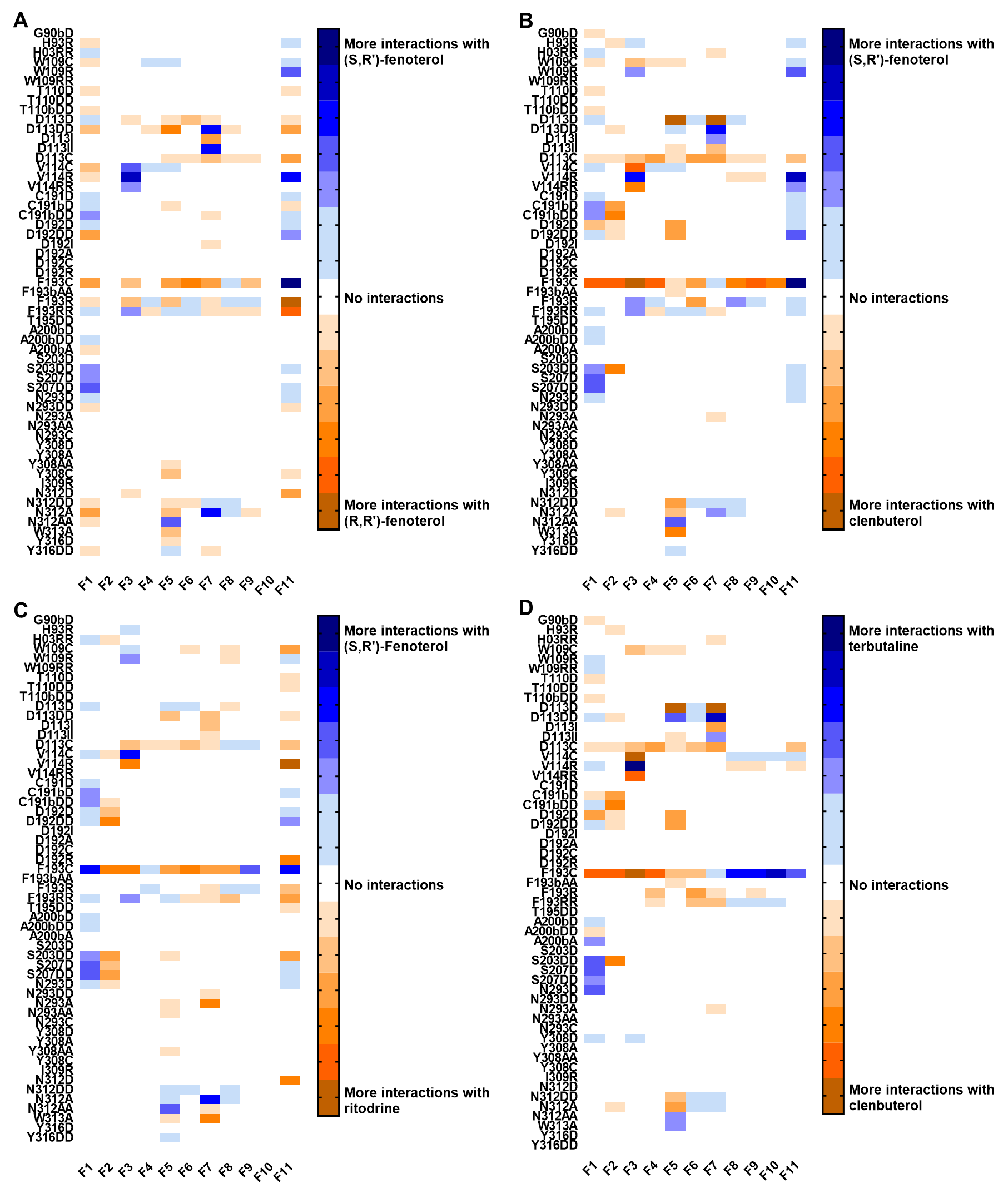


**Supplementary Figure S2.** Biogenic and non-biogenic ligands have distinct interaction fingerprints with the β_2_AR. (*S*,*R*’)-Fenoterol, (*R*,*R*’)-fenoterol, ritodrine, terbutaline, and clenbuterol were docked to inactive structures (3NYA, 3NY8, 5D5B) and active structures (4LDE, 4LDL, 4LDO) of the β_2_AR. From the top 30 poses, ligand interactions were matched with structural features F1-F11 and added for each crystal structure. A. The non-G protein-biased (*S*,*R*’)-fenoterol was compared to the Gα_s_-biased (*R*,*R*’)-fenoterol. B. The biogenic (*S*,*R*’)-fenoterol was compared to the non-biogenic clenbuterol. C. The biogenic (*S*,*R*’)-fenoterol was compared to the partially biogenic ritodrine. D. The partially biogenic terbutaline was compared to the non-biogenic clenbuterol. Interactions are displayed as Residue-interaction type (D- hydrogen bond donor, DD- strong hydrogen bond donor, I- ionic, II- strong ionic, A- hydrogen bond acceptor, AA- strong hydrogen bond acceptor, C- contact, R- arene, RR- strong arene). Residues followed by a b (e.g., G90b) indicate an interaction with the peptide backbone of the corresponding residue.


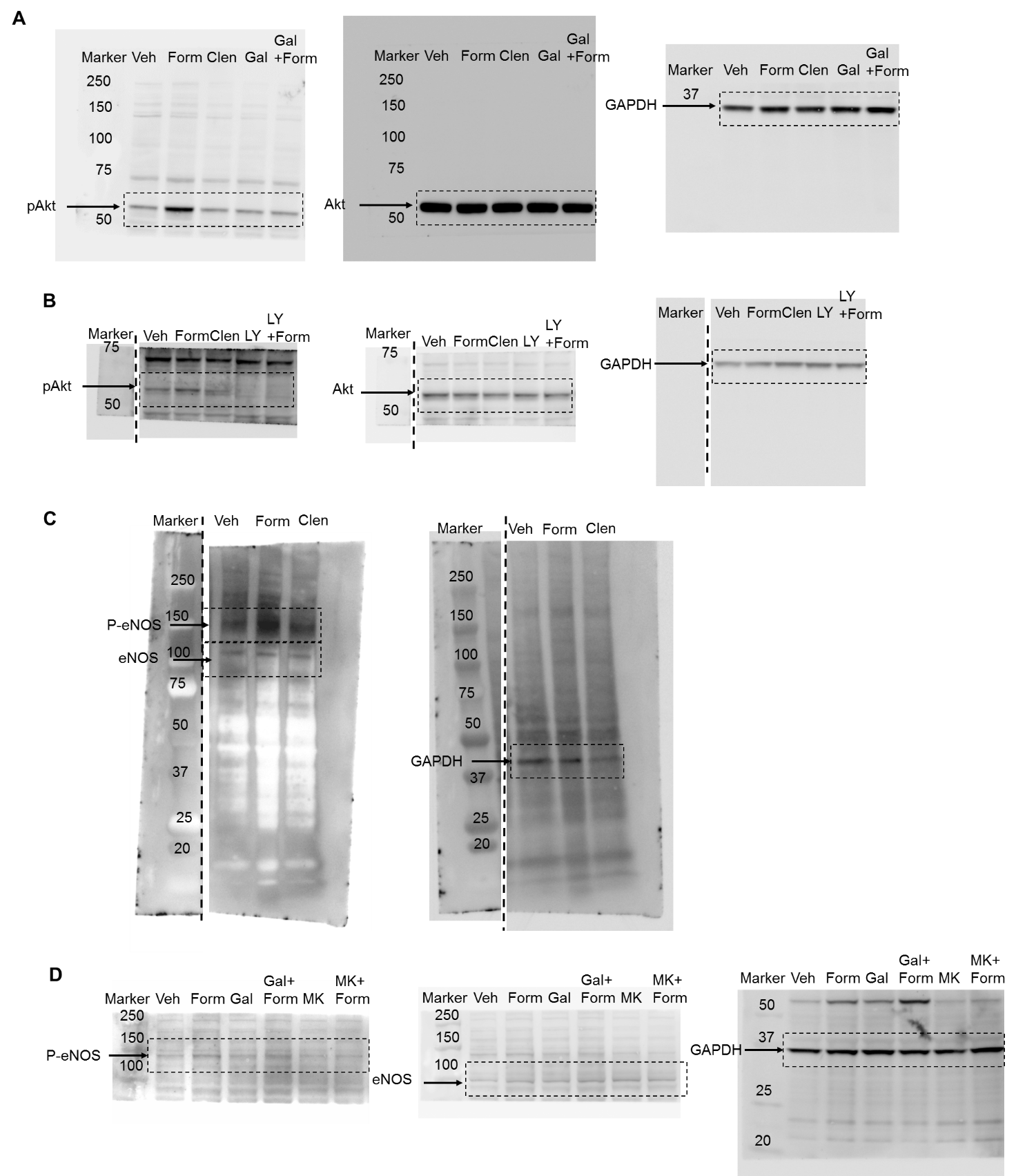


**Supplemental Figure 3.** Uncropped lanes from representative blots. The uncropped lanes from figures 2A (A), 2B (B), 3A (C), and 3B (D) are shown. Bands of interest have a dashed box and are labeled, and lanes are labeled. Unused lanes are cropped, and cropping between lanes is marked by a dotted line. Veh- 0.5% DMSO; Form- 30 nM formoterol; Clen- 30 nM clenbuterol; Gal- 100 nM gallein; LY- 10 μM LY294002; MK- 100 nM MK2206.
